# Contrasting nutrient retention in alpine soils: the role of soil microbiome in phosphorus and nitrogen mobility in scree and meadow environments

**DOI:** 10.1093/femsec/fiag008

**Published:** 2026-02-03

**Authors:** Eva Kaštovská, Michal Choma, Petr Čapek, Jiří Kaňa, Karolina Tahovská, Lenka Čapková, Jiří Kopáček

**Affiliations:** Faculty of Science, Department of Ecosystem Biology, University of South Bohemia in České Budějovice, Branišovská 1760, 37005 České Budějovice, Czech Republic; Faculty of Science, Department of Ecosystem Biology, University of South Bohemia in České Budějovice, Branišovská 1760, 37005 České Budějovice, Czech Republic; Faculty of Science, Department of Ecosystem Biology, University of South Bohemia in České Budějovice, Branišovská 1760, 37005 České Budějovice, Czech Republic; Faculty of Science, Department of Ecosystem Biology, University of South Bohemia in České Budějovice, Branišovská 1760, 37005 České Budějovice, Czech Republic; Biology Centre of the Czech Academy of Sciences, v.v.i., Institute of Hydrobiology, Na Sádkách 7, 37005 České Budějovice, Czech Republic; Faculty of Science, Department of Ecosystem Biology, University of South Bohemia in České Budějovice, Branišovská 1760, 37005 České Budějovice, Czech Republic; Faculty of Science, Department of Ecosystem Biology, University of South Bohemia in České Budějovice, Branišovská 1760, 37005 České Budějovice, Czech Republic; Biology Centre of the Czech Academy of Sciences, v.v.i., Institute of Hydrobiology, Na Sádkách 7, 37005 České Budějovice, Czech Republic

**Keywords:** alpine area, scree soils, nutrient leaching, phosphorus solubilization, nitrogen, ion exchange resin trap

## Abstract

Alpine catchments encompass heterogeneous soil habitats with varying roles in nutrient cycling. While undeveloped till soils in scree areas are hotspots for nitrate and phosphate leaching, vegetated alpine meadow soils rather efficiently retain nutrients. This study examines the role of microbial communities in nutrient mobilization and retention, beyond the effects of abiotic soil properties. We compared the chemical, microbial, and functional characteristics of scree and meadow soils in four high-elevation catchments of the Tatra Mountains in Central Europe. Despite their lower organic matter content and microbial biomass, scree soils exhibited high concentrations of mobile nitrate and phosphate, low phosphate sorption ability, and significantly greater phosphorus leaching. Their microbiomes were distinct and enriched with pioneer taxa, including lichenized fungi, oligotrophic bacterial lineages (e.g. AD3 and Eremiobacteria), and saprotrophic fungi that specialize in the recycling of microbial necromass. These microbiomes exhibited high biomass-specific activities related to nutrient mobilization. In contrast, meadow soils supported larger microbial communities dominated by fungi with strong plant associations and functional traits that enhance nutrient retention. Our findings demonstrate that soil microbiota actively control nitrogen and phosphorus mobility by acting as either accelerators (in vegetation-free scree areas) or buffers (in meadows) of nutrient leaching from alpine soils.

## Graphical Abstract

**Graphical Abstract:**
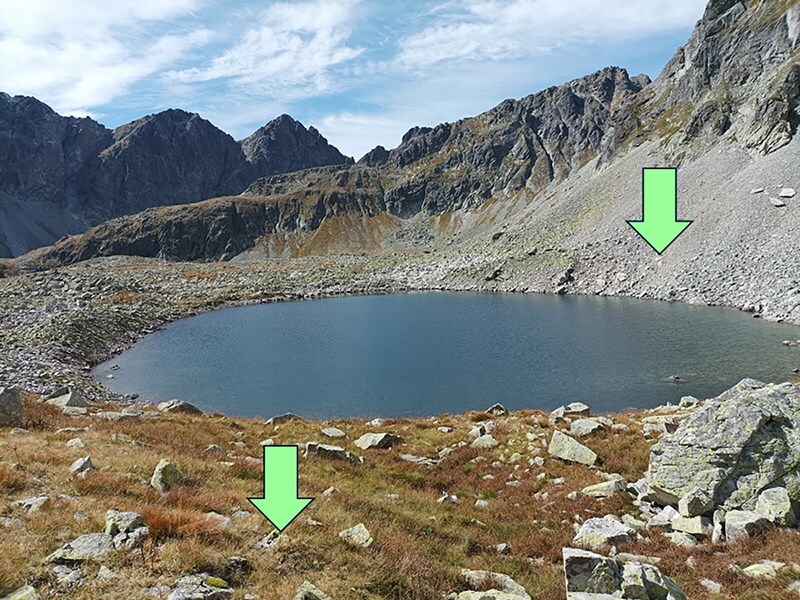
Microbes in alpine soils act as either nutrient accelerators or buffers, driving contrasting nitrogen and phosphorus retention in barren screes and vegetated meadows.

## Introduction

Alpine ecosystems feature complex topography associated with diverse microclimatic regimes that influence soil formation processes, including weathering, erosion, and material transport (Ford et al. 2013, Ernakovich et al. 2014). These conditions create significant local habitat heterogeneity, ranging from bare rock and raw regolith to weathered material buried in scree areas (loose rock fragments covering mountain slopes and accumulated by periodic rockfalls), which form undeveloped scree soils (Kaňa et al. 2023); to rocky, shallow soil mantles (Zinger et al. 2011, Egli et al. 2014) stabilized by vegetation cover and dense root networks, forming meadow soils (Körner 2003). These habitats harbor diverse microbiomes that fundamentally regulate element cycling, soil development, carbon (C) storage, and plant establishment at the local scale. These microbiomes also influence the leaching of organic and mineral nutrients into waters at the regional scale. Microbiomes adapt over time to changes in edaphic conditions and vegetation cover that occur during soil development (Ettema and Wardle 2002, Wardle et al. 2004, Fierer and Jackson 2006, Waldrop et al. 2006), as well as to ongoing climate change (Donhauser and Frey 2018, Moravcová et al. 2023, Broadbent et al. 2024). These adaptations can impact the functioning of mountain ecosystems (Edwards et al. 2007, Ernakovich et al. 2014, Moser et al. 2019).

Among other ecosystem services, mountain regions are important sources of drinking water. The water quality in these remote, high-elevation areas depends, in addition to direct atmospheric deposition and climate, on biogeochemical processes occurring in their catchment areas (Moser et al. 2019). The relative importance of these factors can vary across different regions of the world (Scholz and Brahney 2022). In general, chronically increased atmospheric deposition of inorganic nitrogen (N) (Galloway et al. 2008, Bobbink et al. 2010, Fowler et al. 2013) and, in some regions, phosphorus (P) (Brahney et al. 2015, Scholz and Brahney 2022), together with ongoing warming (Pepin et al. 2015, IPCC 2021), pose major challenges for alpine ecosystems. The combined effect of enhanced nutrient input and longer growing seasons increases terrestrial productivity, alters vegetation and soil microbiome communities, promotes organic matter decomposition and mineralization, and ultimately enhances N and P leaching into receiving waters (Kopáček et al. 2015a, Brahney et al. 2015, Scholz and Brahney 2022).

However, the extent to which these general trends affect individual alpine freshwater systems depends strongly on the specific characteristics of their catchment areas—particularly the composition and spatial coverage of different soil habitats. These habitats are the main sources of major ions and nutrients entering alpine lakes, originating from rock weathering and soil microbial activity, with a contribution from atmospheric input (Moser et al. 2019). The predominant habitat types in the alpine zone, scree areas with undeveloped scree soils representing an early-stage, poorly developed substrate, and areas with more developed alpine meadow soils, differ significantly in their chemical properties (Kopáček et al. 2004, 2015a, 2021), which in turn influence their nutrient mobilization and retention capacity. In particular, scree soils are important sources of ions such as bicarbonate, calcium, sulfate, and especially mineral nutrient forms (nitrate and phosphate), which are leached into waters (Kopáček et al. 2004, Kaňa et al. 2011, 2023). The establishment of plants, along with further soil development and the accumulation of organic matter, increases the ability of meadow soils to bind N and P, increase their stocks, and leach more dissolved organic matter than scree soils (Egli et al. 2012, Kaňa et al. 2011). Recent changes in the quality of catchments with different proportions of soil habitats suggest that the difference in behavior between undeveloped scree soils and more organic-rich meadow soils is further accentuated by climate change (Brahney et al. 2015, Kopáček et al. 2021, Scholz and Brahney 2022).

Differences in nutrient speciation, availability, and mobility between habitat types are due not only to overall nutrient stocks or soil developmental stage, but also to differences in microbial assemblages. Insights into differences between soil microbiomes in alpine scree and meadow habitats can be drawn from studies conducted along chronosequences in glacier forefields in alpine and (sub)arctic regions (Bernasconi et al. 2011, Brankatschk et al. 2011, Goransson et al. 2011, Donhauser and Frey 2018, Wojcik et al. 2020, Luláková et al. 2023) and along elevation gradients (Margesin et al. 2009, Sundqvist et al. 2013, Shen et al. 2015, Kotas et al. 2018, Looby and Martin 2020). These studies indicate that initial substrates such as glacial till, that are poor in organic matter and available nutrients (Bernasconi et al. 2011, Göransson et al. 2016, Wojcik et al. 2020), are primarily colonized by autotrophic and N-fixing bacteria (Schmidt et al. 2008, Luláková et al. 2023) along with relatively simple heterotrophic assemblages (Bardgett and Walker 2004, Bardgett et al. 2007). These pioneer communities rely on atmospheric inputs, N fixation, and sparse carbon sources such as allochthonous organic matter, lichens and early mosses (Bardgett et al. 2007). Their metabolic activity contributes to initial nutrient cycling (Mandolini et al. 2025) and directly enhances physical and chemical weathering of rocks, playing a key role in early soil formation (Bernasconi et al. 2011, Wojcik et al. 2020). With the establishment of vascular plants, the quantity and quality of organic matter input through plant litter and root exudates increase considerably (Schulz et al. 2013). This organic enrichment supports the development of more complex microbial networks, including fungal-based trophic interactions and mutualistic plant–microbe associations (Nemergut et al. 2005, Bardgett et al. 2007, Margesin et al. 2009, Kotas et al. 2018). Although contrasts between scree and meadow soils broadly mirror early- and late-stage alpine soil development, it remains unresolved whether and how consistently are microbial community traits aligned with nutrient mobility across such naturally co-varying gradients of soil chemistry, organic matter accumulation, and hydrology. Based on these findings, we suggest that the early-successional scree soil microbiome contains numerous microbial groups that mobilize nutrients from mineral substrates and atmospheric inputs, such as P-solubilizing bacteria, N fixers, and potentially nitrifiers, which may be favored under conditions of low organic carbon and limited competition for ammonium from heterotrophs or plants. However, due to low microbial biomass, the absence of plant uptake and limited long-term storage in organic matter, scree soils cannot retain these nutrients effectively. The transition to larger, more functionally diverse, fungal-rich microbial communities closely linked to plant roots then improves nutrient retention and recycling in more developed alpine meadow soils. Unlike chronosequence or elevation-gradient studies that infer microbial effects indirectly from soil age or climate, our approach focuses on functional consistency: whether microbial traits expected to promote N and P mobilization or retention systematically co-occur with independent chemical indicators of nutrient mobility.

The aim of this study was to identify the microbial footprint behind the contrasting abilities of scree and meadow soils to mobilize/retain nutrients, and thus their different contributions to terrestrial export of N and P to aquatic environments. Rather than testing whether scree and meadow soils differ per se, we address whether differences in nutrient mobility are consistently accompanied by microbial traits that theoretically promote nutrient mobilization or retention. To do this, we integrated the chemical properties of scree and meadow soils with microbial traits in four high-elevation alpine catchments in the granodiorite massif of the Tatra Mountains (Central Europe). For P mobilization, we identified P-solubilizing bacteria, estimated their relative abundance within the microbiome, measured phosphatase activity, and quantified available P concentration, soil ability to retain phosphate and P leaching from soils in the field using IER traps. For N, we quantified key functional genes related to N-fixation and nitrification, examined the taxonomic composition of associated microbial groups, and measured net N mineralization and nitrification under controlled conditions. We propose the following hypotheses: **H1:** In line with lower phosphate retention ability and higher mineral N and P availability, scree soil microbiomes are smaller and less complex but enriched in P-solubilizing and nitrifying microbes, with higher biomass-specific activities for N and P mobilization. **H2:** Meadow soils host larger, more functionally diverse microbial communities with higher fungal contributions, strongly shaped by plant associations, exhibiting C-mining activities and enhanced nutrient retention.

## Materials and methods

### Study sites and soil collection

The experimental area is located in the central part of a granodiorite massif of the Tatra Mountains (49.2°N, 20.2°E), Slovakia, Central Europe. On the basis of previous studies (Kaštovská et al. 2022, Kaňa et al. 2023), we have selected four small catchments of glacial lakes: Vyšné Wahlenbergovo, Pusté, Ladové, and Velké Hincovo, hereinafter referred to as VW, PU, LA, and VH, respectively (Table 1). The catchments were located in the upper parts of geomorphologically isolated, parallel valleys above 1900 m a.s.l. The valleys are similarly south-facing and within 10 km apart. More than 30% of the study catchments were covered by scree deposits. Scree contains isolated small ‘pockets’ of weathered substrate, the unvegetated scree soils that are hidden under and between the rock fragments without forming a continuous layer. The estimated amount of scree soil (<2 mm fraction; dry weight) ranged from 4 to 16 kg m^−2^, with an average of 9 kg m^−2^ in the upper 0.5 m thick layer. The scree soils represent pioneer habitats with poor soil structure and low organic matter content (Kopacek et al. 2021, Kaňa et al. 2023). The meadow soils are predominantly shallow Leptosols (WRB classification), with a dark, 10–15 cm deep organomineral A horizon formed directly on the cracked and slightly weathered bedrock. A basic description of the catchments including soil temperatures measured continuously (hourly) at the sampling points in 2021 using HOBO® UTBI-001 sensors (Onset Computers, USA) with an accuracy of ±0.2°C is given in Table 1.

**Table 1:** Characteristics of the four catchments from which the scree and meadow soils pair originated.

Catchment	Abbr.	Altitude of lake water level (m a.s.l.)	Catchment area (ha)	% scree coverage	Soil type	Soil temperature in 5 cm depth (°C), min/max/MAST	Ice days (*T*_min_ < 0 °C) per year
Vyšné Wahlenbergovo	VW	2157	32	46	Scree	-6.6/16.0/0.7±5.0	213
					Meadow	-6.0/20.1/2.3±5.6	190
Pusté	PU	2056	20	43	Scree	-10.5/21.6/0.9±6.6	203
					Meadow	-7.1/20.6/3.6±5.8	131
Ladové	LA	1925	13	64	Scree	-5.9/23.4/2.3±5.9	159
					Meadow	-1.1/16.1/3.6±4.8	117
Velké Hincovo	VH	1945	127	32	Scree	-4.7/21.3/2.4±5.1	223
					Meadow	-1.7/15.6/2.8±4.2	148

Lake surface elevations, catchment areas and their scree cover (%), minimum, maximum and mean annual soil temperature (MAST) measured at 5 cm depth between 15. 9. 2021 and 15. 9. 2022, and the number of days per year when the temperature fell below 0 °C.

The soils were collected in September 2021, i.e. in the late growing season in the Tatra Mountains. In each of the four catchments, three composite soil samples were taken from three different alpine meadows and three scree fields. All sampling sites were close to lakes and had similar elevation. Each composite sample of the meadow soil consisted of five subsamples taken with a spade from a depth of 0–15 cm. Each composite sample of the scree soil consisted of at least five subsamples taken with a small shovel from a scree field in an area of ∼50 m^2^ near the sampled meadow. In addition, small stones (<2 cm) were collected from the scree field in the same area and used to isolate P-solubilizing bacterial strains (details below). As a part of the sampling campaign, three mixed soil samples from meadows and scree fields and three mixed stone samples from scree fields were collected in each of the four catchments. On the day of sampling, the fresh samples were sieved through a 5 mm mesh sieve, and a 5–10 g subsample was immediately frozen at −20°C and preserved for microbial DNA extraction and enzymatic activity analysis from each sample. The remaining samples were stored in the dark at 4°C for one week until further processing.

### Soil physico-chemical and biochemical analyses

Soil samples dried at 60°C for 2 days and milled were used to determine concentrations of total organic C, N, and P. The C and N concentrations and their isotopic composition were analysed with NC Elemental analyzer (ThermoQuest, Germany) connected to an isotope ratio mass spectrometer (IR-MS Delta X Plus, Finnigan, Germany). The measured ratio of ^13^C/^12^C was expressed as δ^13^C using a Vienna PDB standard and the ^15^ N/^14^ N ratio was expressed as δ^15^ N using N_2_ in air as the standard. Total P was measured colorimetrically by the ammonium molybdate-ascorbic acid method on a flow injection analyzer (FIA, Lachat QC8500, Lachat Instruments, USA) after perchloric acid digestion (Kopáček and Hejzlar 1995). The soil pH was determined using a glass electrode in a soil–distilled water mixture (1 : 5, w/v).

Concentrations of C, N, and P in soil microbial biomass (MBC, MBN, and MBP, respectively) were assessed by chloroform fumigation-extraction method (Brookes et al. 1982, 1985, Vance et al. 1987a). Fresh soil samples were extracted both directly and after 24-h fumigation in ethanol-free chloroform with 0.5 M K_2_SO_4_ (1 : 4, w: v) for MBC and MBN and with 0.5 M NaHCO_3_ (1 : 15, w: v) for MBP, respectively. In case of MBP, another set of fresh samples with a known amount of added internal standards (KH_2_PO_4_) was extracted to correct the further calculations for P sorption/extraction efficiency. Phosphorus in sodium bicarbonate extract from fresh soil corresponds to the bioavailable P known as Olsen P (Olsen and Sommers 1982). The internal standard recovery was further used to estimate the soil’s ability to retain phosphate mainly due to its sorption on Fe and Al hydroxides. Concentrations of organic C and total N in the potassium sulfate extracts were analyzed using a TOC-L analyzer with the total N measuring unit TNM-L (Shimadzu, Japan). All bicarbonate extracts were acidified with H_2_SO_4_ and analyzed for reactive P concentrations colorimetrically (Brookes et al. 1982). The MBC, MBN, and MBP were calculated as differences in the concentrations of organic C, total N, and reactive P in soil extracts before and after fumigation. The values were corrected for extraction efficiencies using k_EC_ = 0.38 (Vance et al. 1987b), k_EN_ = 0.45 (Brookes et al. 1985), and k_EP_ = 0.4 (Brookes et al. 1982). MBP was additionally corrected for recovery of internal P standard.

Concentrations of dissolved organic C (DOC), total dissolved N (DN), ammonium N (NH_4_-N), nitrate N (NO_3_-N), and soluble reactive P (SRP) were measured in soil extracts with distilled water (1 : 10, w/v) shaken an hour on a horizontal shaker. Concentrations of NO_3_-N, NH_4_-N, and SRP in the extracts were measured colorimetrically, using a flow injection analyzer (FIA Lachat QC8500, Lachat Instruments, USA), and DOC and DN were analyzed as described above for organic C and total N concentrations. The water extracts were used to represent the most mobile C, N, and P forms. Unless explicitly stated, all element concentrations in soil reported in this study are given on a dry weight (DW; 105 °C) sample basis.

### Characterization of P mobilization and leaching potential of scree and meadow soils

#### In situ measurement of phosphate leaching by Fe-based ion-exchange resin (IER) traps

We measured annual phosphate leaching from the soils *in situ* for two years, using a hybrid anion resin (Purolite FerrIX A33E) to capture phosphate (PO_4_-P) leached from the soils. The anion resin was pretreated according to Tahovská et al. (2016) and filled in circular traps (an inner diameter of 6 cm and a height of 1 cm). These IER traps were placed horizontally to a depth of about 5 cm in the meadow soil and under the sufficient amount (ca 2 cm depth) of scree soil in the pockets between the boulders to capture phosphate flowing from top to bottom over a corresponding area of 28.3 cm^2^. All the IER traps were replicated 10 times per catchment and replaced once a year not to exceed their capacity. After sampling, the resins removed from the IER traps were washed in demineralized water, transferred to a glass column (3 cm in diameter and 30 cm in height) with ceramic frit and extracted by a repeated elution procedure with a 2% NaOH to obtain 300 ml of the final eluate (Tahovská et al. 2016). The concentration of SRP was measured according to (Murphy and Riley 1962) and corrected for elution efficiency (Tahovská et al. 2016). The amount of the IER-trap P was expressed as annual P flux per g (meadow or scree) soil.

### Basal soil respiration, exoenzymatic C, N, and P-mining, and oxidative activity

Basal soil respiration at 15°C (common daily temperature maxima at 5 cm soil depth in sampling sites during the summer season) was chosen as a general proxy-parameter of soil microbial activity. Fresh soils (10 g) were pre-incubated in 100 ml bottles at 15°C for 10 days to stabilize microbial activity, then sealed airtight and CO_2_ accumulation in a headspace was measured after 24 h using gas chromatography (Agilent Technologies, Santa Clara, CA, USA).

Activities of hydrolytic and oxidative enzymes, providing an insight to soil organic matter dynamics and microbial needs for organic nutrients (Chen et al. 2025), were determined using microplate fluorometric and colorimetric assays depending on added substrate (i.e. forming either fluorescent or colored reaction product) (German et al. 2011). To determine hydrolytic enzyme activities, 0.5 g of thawed soil was suspended in 50 ml of distilled water and sonicated for 4 min to break up the soil aggregates. The soil suspension (200 µl) was spiked with 50 µl of a methylumbelliferyl substrate solution specific to class of enzymes to be determined—β-glucosidase (BG), cellobiosidase (CEL), phosphatase (PHO), and chitinase (CHIT). For the determination of leucine aminopeptidase (LAP), 200 µl of soil suspension was added to 50 µl of 7-aminomethyl-4-coumarin substrate solution (Bárta et al. 2014). The plates were incubated in the dark at 15°C for 2 h. Fluorescence was quantified at an excitation wavelength of 365 nm and an emission wavelength of 450 nm using the INFINITE F200 microplate reader (TECAN, Germany). All hydrolytic enzymatic activities were summed and the proportions of C mining (BG and CEL), N mining (LAP and CHIT), and P mining (PHO) were calculated to document object of microbial nutrient mining (Sinsabaugh et al. 2009) and to compare the potential for nutrient mobilization in scree and meadow habitats. The oxidative activity was measured to characterize the potential of the soils to catalyze breakdown of recalcitrant polyphenolic and lignin-like organic compounds, using L-3,4-dihydroxyphenylalanine (L-DOPA) as a substrate and hydrogen peroxide addition (Bach et al. 2013). Absorbance was measured at a wavelength of 450 nm after incubation of the plates for 18 h at 25°C. The ratio of the recalcitrant C degrading oxidative activity to activities of labile C degrading hydrolases (BG+CEL) was used as an indicator of the availability and recalcitrance of soil C pool (Sinsabaugh and Follstad Shah 2011).

### Microbial DNA, bacterial and fungal abundance

Total microbial DNA was extracted from 0.25 g of defrosted soil using a DNEasy PowerSoil Pro Kit (Qiagen, Germantown, MD, USA) according to manufacturer protocol. Bacterial and fungal marker genes were quantified, as described previously (Tahovská et al. 2020). Briefly, primer pairs 341F/534R and nu-SSU-0817–5′/nu-SSU1196-3′ were used to target bacterial 16S rRNA and fungal 18S rRNA genes, respectively (Borneman and Hartin 2000, Muyzer et al. 1993) and their abundance was quantified on the StepOne™ Real-Time PCR System (Life Technologies, USA) using a serial dilution of the known amount of a purified PCR product obtained from plasmids containing Escherichia coli and Aspergillus niger rRNA genes as standards in the case of bacteria and fungi, respectively. The sum of 16S copies divided by 18S copies was used as the bacteria-to-fungi (B/F) ratio.

### Composition of bacterial and fungal microbiomes

The composition of microbial communities was characterized by barcoded amplicon sequencing using the Illumina NovaSeq 6000 System using Nextera™ technology (performed by SEQme s.r.o., Czech Republic). The analysis targeted the bacterial V4 region (primers 515f/806r; Caporaso et al. 2011) and the fungal ITS region (primers ITS1F/ITS4R; White et al. 1990).

The detailed description of raw reads processing is available on https://github.com/chomic-kbe/tatry_scree. Briefly, in case of bacteria, primers were detected and trimmed from raw reads with cutadapt (Martin 2011), *dada2* v.1.36.0 (Callahan et al. 2016) was used to perform quality filtering (maxEE=1, rm.lowcomplex = 8), ASV table generation and taxonomy annotation using SILVA 138.2 (Quast et al. 2013). One sample did not yield sufficient amount of sequence reads and was discarded from further analyses. Processing of fungal ITS reads was based on recommendations given by Pauvert et al. (2019). Only forward reads were preprocessed using *dada2*. ASV sequences resulting from quality filtered (maxEE=2, rm.lowcomplex=6) reads were taxonomy annotated by BLAST through PipeCraft (Anslan et al. 2017) against UNITE version 10 (Abarenkov et al. 2024). The >98.5% identity was considered sufficient to accept assignment of UNITE Species Hypotheses (SHs; Kõljalg et al. 2020). Assignments with <98.5% identity were accepted at the best reliable taxonomic resolution according to lineage-specific thresholds proposed in Table S1 of Tedersoo et al. (2022). Read abundance of ASVs assigned to the same SH were summed into one value per each SH. Remaining ASVs were unedited. Fungal lifestyle was assigned to genera with help of FungalTraits (Põlme et al. 2021). ASVs with total abundance < 10 reads across the respective datasets were discarded. Diversity indices (Chao1 and Shannon) were calculated from datasets rarefied to minimal sample sequencing depth (18 114 and 5508 for bacteria and fungi, respectively).

### Characterizing N transformation and mobilization potential

Genetic potential for the certain processes within N cycling were assessed by quantification of respective functional genes, specifically, *nifH* for N fixation (enriching the habitat with N), *AmoA-A* and *AmoA-B* for ammonium oxidation to nitrite by archaea and bacteria (the first step of nitrification creating mobile/leachable nitrate), and *nirS* and *nirK* genes for denitrification (gaseous N losses, potentially reducing mobile nitrate in soils) using qPCR. Additionally, the potential rates of net N mineralization (ammonification) and nitrification were measured as the temporal differences in the concentrations of NH_4_-N and NO_3_-N, respectively, in the 0.5 M K_2_SO_4_ soil extracts between the 7th and 14th day of lab incubation at 15°C on a flow injection analyzer (FIA Lachat QC8500, Lachat Instruments, USA). PICRUSt2 was used to predict the relative abundance of bacterial (and archaeal) ASVs potentially participating in ammonium oxidation by selecting those who were predicted to possess genes for ammonia monooxygenase (EC 1.14.99.39) (Douglas et al. 2020).

### Isolation and characterization of P-solubilizing bacteria from rock surfaces, scree, and meadow soils

The PSB were isolated from scree and meadow soils and stone samples as follows: for soils, 1 g was suspended in 50 ml of sterile 0.9% NaCl, sonicated (1 min), and shaken (30 min, 100 r/m). Serial dilutions (10⁻¹–10⁻³) were plated on Pikovskaya (PKV) agar in 3 replicates of 100 µl per dilution (Nine plates per sample). Stones for PSB isolation were placed in a 1000 ml beaker up to the 500 ml mark, filled with 500 ml of sterile 0.9% NaCl, sonicated (1 min), and shaken (30 min, 100 r/m). Only clean stones without mosses or lichens were used. Serial dilutions (10^0^–10⁻²) were plated on PKV agar as above (Nine plates per sample). PKV agar media (Pikovskaya 1948) consisted of 10 g glucose, 5 g Ca_3_(PO_4_)_2_, 0.5 g (NH_4_)_2_SO_4_, 0.2 g NaCl, 0.2 g KCl, 0.5 g yeast extract, 0.002 g MnSO_4_, 0.002 g FeSO_4_, and 15 g agar in one L distilled water. The only P source in PKV agar is insoluble apatite Ca_3_(PO_4_)_2_, which is responsible for the milky color of the medium. Bacterial colonies capable of solubilizing P dissolve apatite creating a transparent ‘halo’ zone. All inoculated PKV agar plates were incubated at 20°C for 7 days. After incubation, all colonies surrounded by the halo zone were transferred to a new sterile PKV agar plate to confirm the formation of the halo zone in pure culture.

To identify the isolates, DNA was extracted from the clear cultures using the DNEasy Ultra Clean Microbial Kit (Qiagen, Germantown, MD, USA) according to the manufacturer’s protocol. The 16 s rRNA gene was amplified using primers 9bfm (GAGTTTGATYHTGGCTCAG) and 1512uR (ACGGHTACCTTGTTACGACTT) (Mühling et al. 2008). The products were purified using QIAquick PCR purification KIT (Qiagen, Germantown, MD, USA) and sequenced in the forward and reverse direction using the Sanger method (performed by SEQme s.r.o, Czech Republic). The reads were assembled and aligned in Geneious Prime 2025.1.2 (Biomatters Ltd., Auckland, New Zealand) and similarity searches against the nr/nt nucleotide and RefSeq Loci 16S databases at NCBI were performed.

To confirm capability of P solubilization, at least one isolate of each retrieved 16S rRNA gene variant was incubated in PKV liquid media, which had the same composition as PKV agar media except for agar. Bacteria were first pre-incubated in liquid nutrient media (5 g casein peptone, 2.5 g yeast extract and 1 g glucose per 1 l) on shaker (135 r/m) for two days at lab temperature. 100 µl of inoculum (in concentration equivalent to optical density of 0.6 at 600 nm) was then transferred to sterile PKV liquid media and shaken in incubator for 7 days at 25°C and 135 r/m in three replicates. After the incubation, the media were filtered (0.45 µm glass-fibre filter), and pH and concentrations of SRP were determined similarly as in soil water extracts.

Only isolates that significantly increased SRP or decreased pH in PKV liquid media compared to sterile controls (Dunnett test) were considered as PSB. The absence of SRP increase does not necessarily mean absence of P solubilization since the solubilized P may be readily immobilized. Therefore, pH decrease was considered as an indicator of PSB activity too. To estimate relative abundance of PSB in soil bacterial communities, V4 region was extracted from 16S rRNA reads representing all unique (total 37) PSB genotypes using cutadapt and 515f/806r primer sequences. Extracted sequences were directly mapped against a reference database composed of soil bacterial ASVs using USEARCH 11 with global alignment and 99% identity threshold (Edgar 2010). ASVs that were successfully mapped with PSB sequences were further considered as “potential PSB ASVs”.

### Statistical evaluation of the data

The Shapiro–Wilk test was used as the primary diagnostic for residual normality due to its robustness and acceptance for small to moderate sample sizes. The homogeneity of residual variances across groups was assessed using Levene’s test (the *car* package). Where assumptions were not met, log-transformations of the response variable were applied to improve normality and homoscedasticity. Data containing negative values (δ^13^C, net ammonification and nitrification rates) were first shifted to positive values (by subtracting the minimum value and adding one).

To test differences between characteristics of scree and meadow soils, we used linear mixed-effects models (LMMs) fitted by restricted maximum likelihood (REML) via the lmer() function from the *lme4* package (Bates et al. 2015). These models included soil type (meadow *vs* scree) as a fixed effect, and catchment as a random intercept effect, to account for non-independence of samples collected from the same site. The proportion of variance (ICC) explained by the random effect (catchment) relative to the total variance (random effect + residual) was calculated. The significance of fixed effect (soil type) was evaluated using Satterthwaite’s approximation for degrees of freedom and corresponding p-values, as implemented in the *lmerTest* package (Kuznetsova et al. 2017). Model residuals were tested for normality using Shapiro–Wilk test. To facilitate a meaningful comparison of a large number of measured soil characteristics, we visualized them as relative differences between scree and meadow soils. The relative differences were expressed as the decadic logarithm of the response ratio (log Response Ratio) calculated against the common median. A log Response Ratio greater than 0 indicates that a characteristic measured in the specific soil type exceeds the median value calculated across both soil types, and vice versa (approximately corresponding to a t-test). When the log Response Ratio of a given characteristic is close to zero in both soil types, and the error bars representing lower and upper quartiles intersect zero, the difference between the soil types is not statistically significant. Tables of all measured soil parameters for scree and meadow soils in individual catchments, information on data transformation, and statistical evaluation results are provided in the Supplement (Tables S1–S3).

Microbial community data was processed in statistical program R with help of *phyloseq* v. 1.52.0 (McMurdie and Holmes 2013). Except for alpha diversity indices (Chao1, Shannon), all analyses were based on non-rarefied data (McMurdie and Holmes 2014). Effect of soil type and catchment were tested by the PERMANOVA analysis of Bray–Curtis dissimilarities at the ASV and SH/ASV level for bacteria and fungi, respectively (*adonis2*, R package *vegan* v. 2.7–1 (Oksanen et al. 2025). To visualize sample differences, ordination diagrams of non-metric multi-dimensional scaling (NMDS) were plotted. To determine bacterial orders and ASVs and fungal SHs/ASVs with significantly different abundance between meadow and scree soils, differential gene expression analysis (DESeq2) was performed (Love et al. 2014).

To examine multivariate co-variation among soil type, indicators of N and P mobility as response variables, and selected microbial traits as explanatory variables, redundancy analysis (RDA) was conducted using CANOCO for Windows v. 5 (Šmilauer and Lepš 2014). Predictor effects were assessed with 999-permutation Monte Carlo tests, and forward selection was used to identify variables that significantly explained adjusted variation.

## Results

### Temperature regimes and basic physicochemical properties of meadow and scree soils

Both meadow and scree soils had low mean annual soil temperatures (MAST <4°C at 5 cm depth), with soil temperatures remaining below 0°C for at least one third of the year (Table 1). Meadow soils experienced a milder thermal regime, with MAST values 0.5°C–3°C higher, and were warmer in winter (by 0.5°C–4°C) and summer (by 2°C–7°C). They also had 20–70 fewer freezing days. In contrast, scree soils, despite being covered with rock, were exposed to more extreme climatic conditions (Table 1).

**Figure 1:**
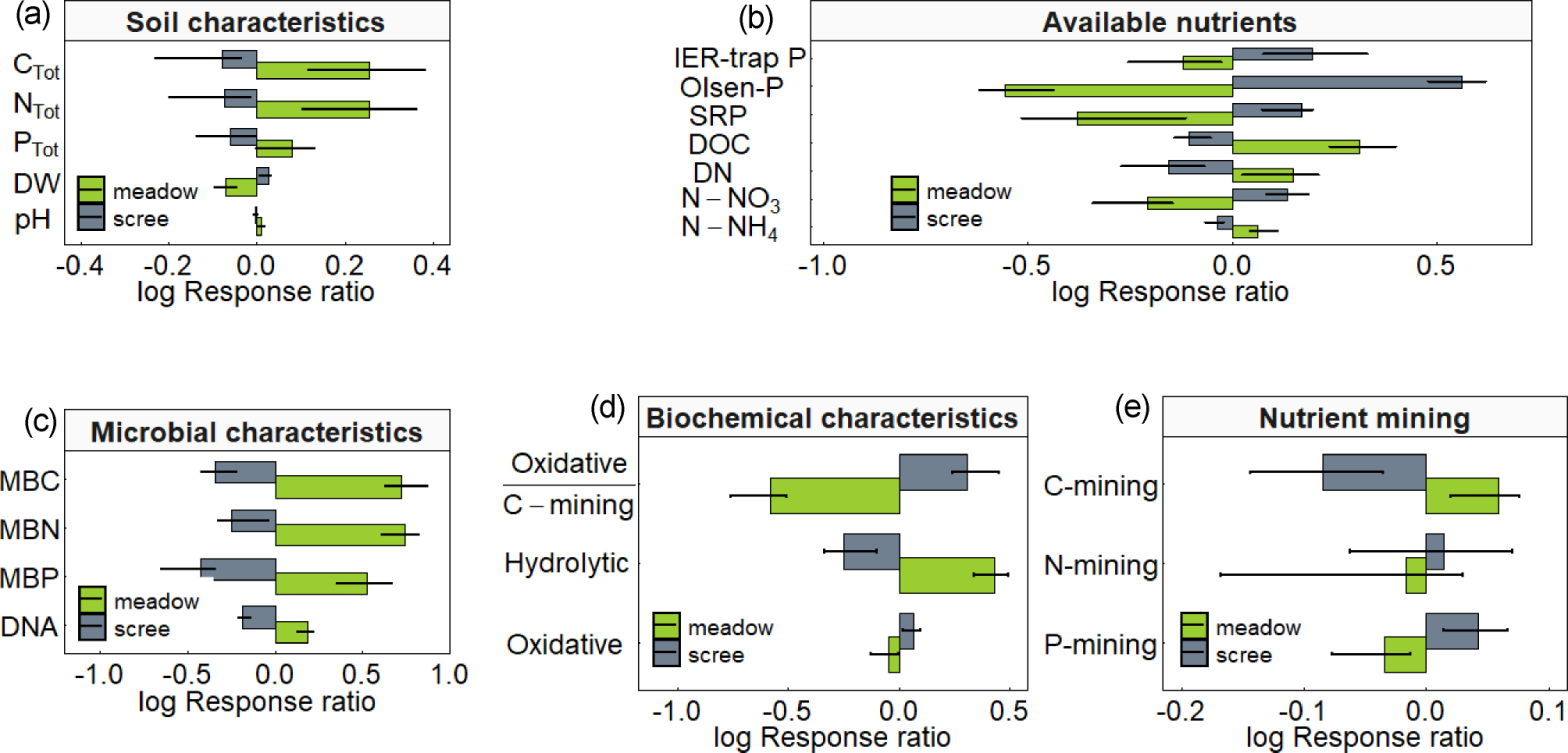
Comparison of basic soil properties in scree and meadow soils. Bars represent decadic logarithms of response ratios normalized to a common median. For each soil property, bars for meadow and scree are shown. Bar height corresponds to the soil-type-specific median of the log response ratio, and error bars indicate lower and upper quartiles. This plot provides an approximate visualization of a one-sample t-test for each property. Larger log response ratios indicate greater deviation of the soil property from the common median across soil types.

Compared to scree soils, meadow soils had on average about twice as high concentrations of organic C, which was positively correlated with soil N concentrations (r = 0.997, *P* < 0.001), and about 27% more total P (Fig. 1a). They also exhibited higher pH and moisture content (i.e. lower DW; Fig. 1a, Table S1). The plant-derived nature of soil organic matter in meadows was reflected by lower ¹³C content and δ¹³C values (−25.96 ± 0.27‰) compared to scree soils (−24.03 ± 0.41‰). In contrast, δ¹⁵N values were similar in both soil types, averaging 3.59 ± 1.08‰ in scree and 4.01 ± 0.82‰ in meadow soils, indicating a common dominant N source.

### Concentration, speciation, and mobility of available N and P

Meadow soils contained more water-soluble DOC and DN than scree soils (Fig. 1b), except in the VW catchment, where both DOC and DN contents were comparably low in scree and meadow soils (Table S1). In scree soils, DN consisted >40%, and in the extreme case of VW even >80%, of mineral N forms, dominated by NO_3_–N (Table S1). Consistently, NO_3_–N concentrations were higher in scree than in meadow soils (Fig. 1b). Together with the lower C/N ratio in water extracts from scree than from meadow soils (10.6 *vs*. 13.3 on average, Table S1), this indicates higher N availability and mobility in scree soils.

Scree soils, despite having almost 30% lower total P concentrations, were characterized by higher P availability and mobility, as reflected by more than twice the concentrations of water-soluble SRP and bioavailable Olsen P compared to meadow soils (Fig. 1b). They also showed, on average, a 20% higher extraction efficiency of the added P standard solution (Table S1), indicating a lower ability to retain mobile phosphate. Consistent with this, *in situ* exposed IER traps captured about twice as much phosphate in scree than in meadow soils (Fig. 1b).

### Soil microbial biomass, respiration, and exoenzymatic activities targeting C, N, and P mining

The organic matter-rich meadow soils contained greater microbial biomass, as reflected by higher MBC, MBN, MBP, and soil DNA (Fig. 1c). Their microbiome was more dominated by fungi (both per DNA and per gram of soil) and had a higher biomass C/N ratio compared to scree soils, whereas bacterial abundances were similar in both soil types (Table S2). Meadow soils also exhibited higher respiration rates and overall hydrolytic activity (Fig. 1d), driven by increased activities of most measured hydrolytic exoenzymes, including glucosidase, cellulose, chitinase, and phosphatase, with the exception of Leu-aminopeptidase (Table S3).

The proportion of C-mining enzymes in total hydrolytic activity was higher in meadow soils, while scree soils showed a higher relative investment in P-mining (Fig. 1e). Exceptions were the PU and VW sites, where these differences were not significant (Table S3). Leu-aminopeptidase activity, which releases simple amino acids as the final step of protein depolymerization, and the relative contribution of N-mining to total hydrolytic activity were comparable between soil types (Fig. 1e). The ratio of oxidative to C-mining hydrolytic activities was about six times higher in scree soils (4.28 ± 2.15) than in meadow soils (0.64 ± 0.51) (Fig. 1d).

### N cycling and mobilization: quantification of functional genes, functional annotation of microbiome composition, and net nitrification

Scree soils, which had smaller microbial biomass, contained lower numbers of *nifH, nirK*, and *nirS* gene copies per gram of soil, indicating reduced potential for N fixation (input of external N) and denitrification (gaseous N losses) compared to meadow soils (Table S2). In contrast, the number of *AmoA-B* gene copies, representing bacterial nitrifiers, was similar between soil types, while *AmoA-A* copies, associated with archaeal nitrifiers, were significantly more abundant in scree soils, both per DNA and per gram of soil. This suggests a stronger involvement of Archaea in nitrification in scree soils than in meadows (Table S2).

**Figure 2:**
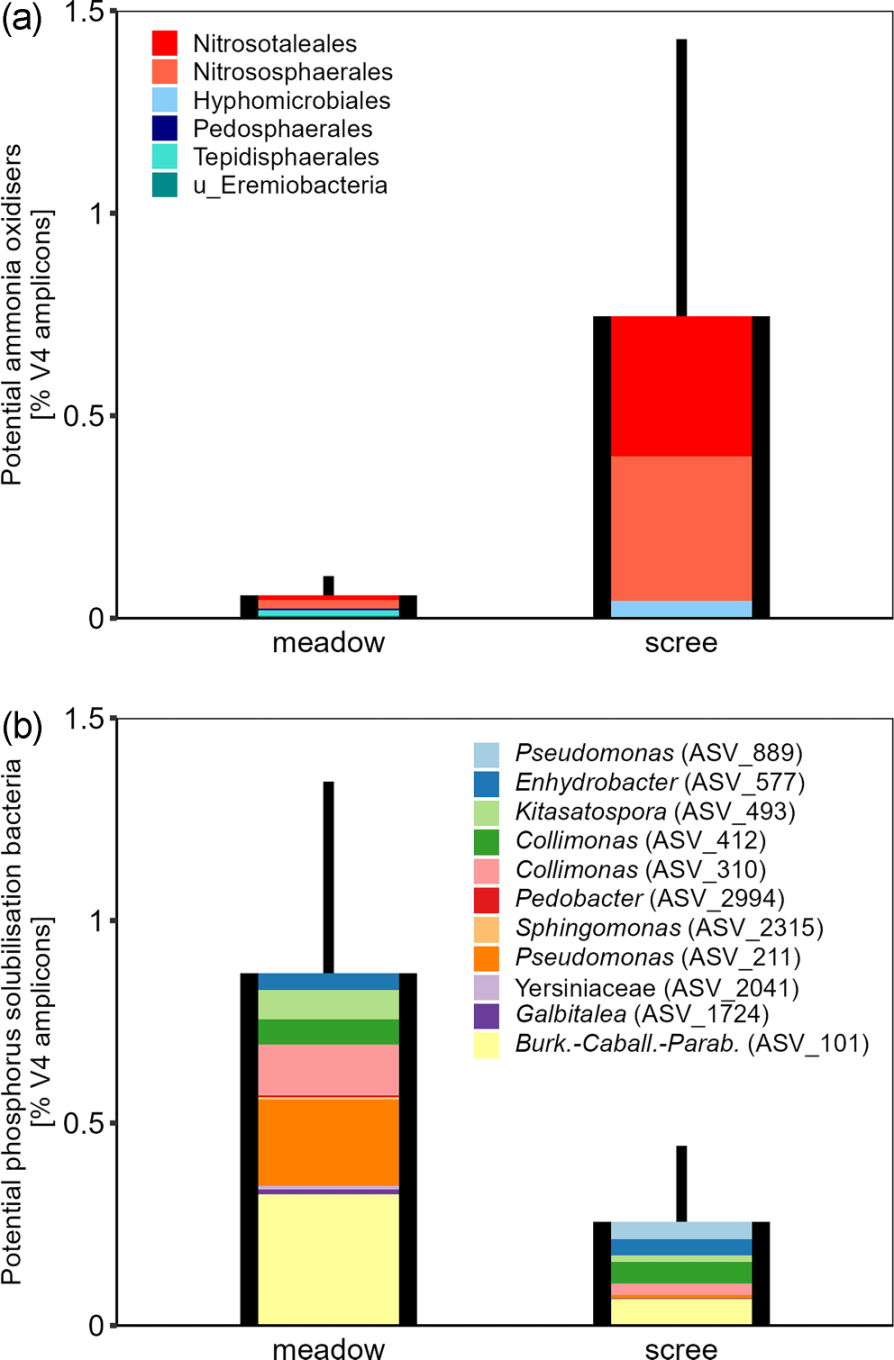
Relative abundance of potential ammonia oxidizers (A) and potential phosphorus solubilizing bacteria (PSB; B) in the prokaryotic communities of scree and meadow soils. (A) Black bars and whiskers show the mean ± SD of the sum of all potential nitrifiers, while the coloured segments indicate contributions of individual orders; red shades denote archaeal taxa. (B) Black bars and whiskers show the mean ± SD of the sum of all potential PSB ASVs, with coloured segments representing individual ASVs.

A similar pattern was observed in predicted nitrification potential from prokaryotic V4 sequencing (Picrust2), which showed higher relative abundances of archaeal nitrifiers (Nitrososphaerales and Nitrosotaleales) in scree microbiomes compared to meadow communities (Fig. 2A; Tables S6 and S7). Despite the lower microbial biomass in scree soils, laboratory incubations revealed similar rates of net ammonification and net nitrification in both soil types (Table S3).

### Phosphorus solubilizing bacteria (PSB)

From soils and scree rocks, we isolated 37 bacterial genotypes showing potential for P solubilization, either by increasing SRP concentration or lowering the pH of Pikovskaya liquid media (Table S4). Most genotypes belonged to Gammaproteobacteria: Burkholderiales—*Burkholderia-Paraburkholderia-Caballeronia* (8) and *Collimonas* (6); Pseudomonadales—*Pseudomonas* (7) and *Moraxella* (1); Enterobacterales—Yersiniaceae (7). We also identified five Actinobacteria genotypes: *Frondihabitans* (3), *Kitasatospora* (1), and *Rhodococcus* (1). Other classes were represented by a single genotype each: Alphaproteobacteria: *Sphingomonas, Bacteroidia: Pedobacter*, and Bacilli: *Paenibacillus*.

Twenty-six genotypes were successfully assigned to bacterial ASVs in the soil microbiome. However, in diverse, species-rich genera (e.g. *Burkholderia-Paraburkholderia-Caballeronia, Collimonas, Pseudomonas*), multiple genotypes corresponded to a single ASV. Consequently, we identified 10 potential PSB ASVs (Table S4). These ASVs were more abundant in meadow soils, both in relative abundance (∼0.9% vs. ∼0.3% in scree soils) and in richness (9 ASVs in meadow vs. 7 in scree soils; Fig. 2B, Table S4).

### Composition of prokaryotic and fungal microbiomes of meadow and scree soils

**Figure 3:**
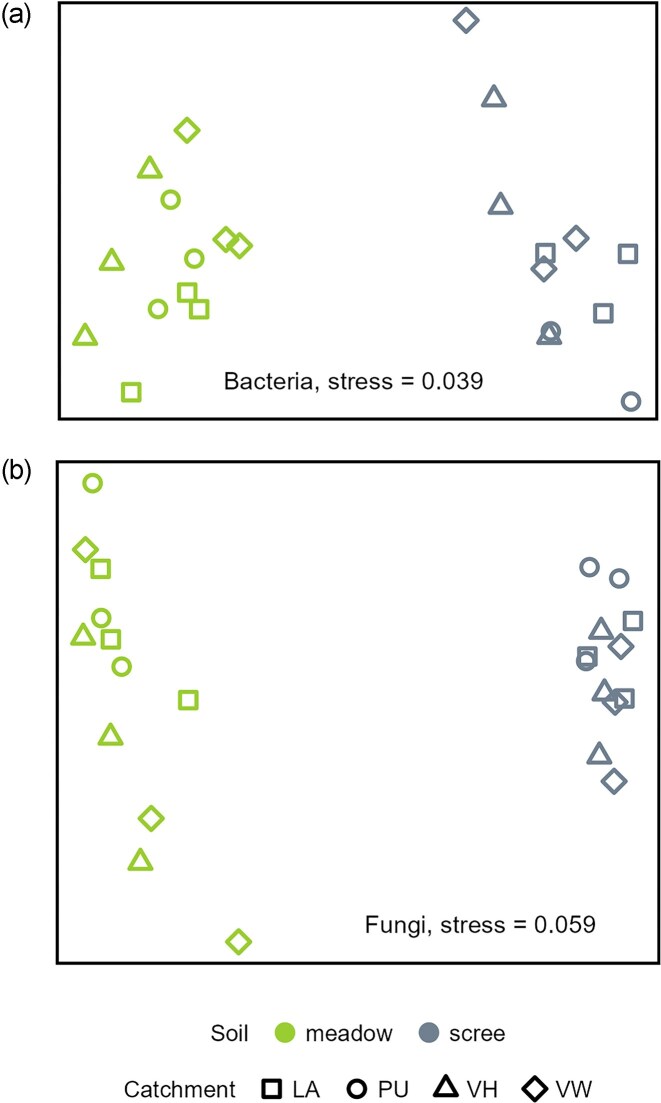
Differences in bacterial (A) and fungal (B) community composition between scree and meadow soils, visualized using NMDS ordination based on Bray–Curtis dissimilarity. Different shapes represent samples from four catchments.

**Figure 4:**
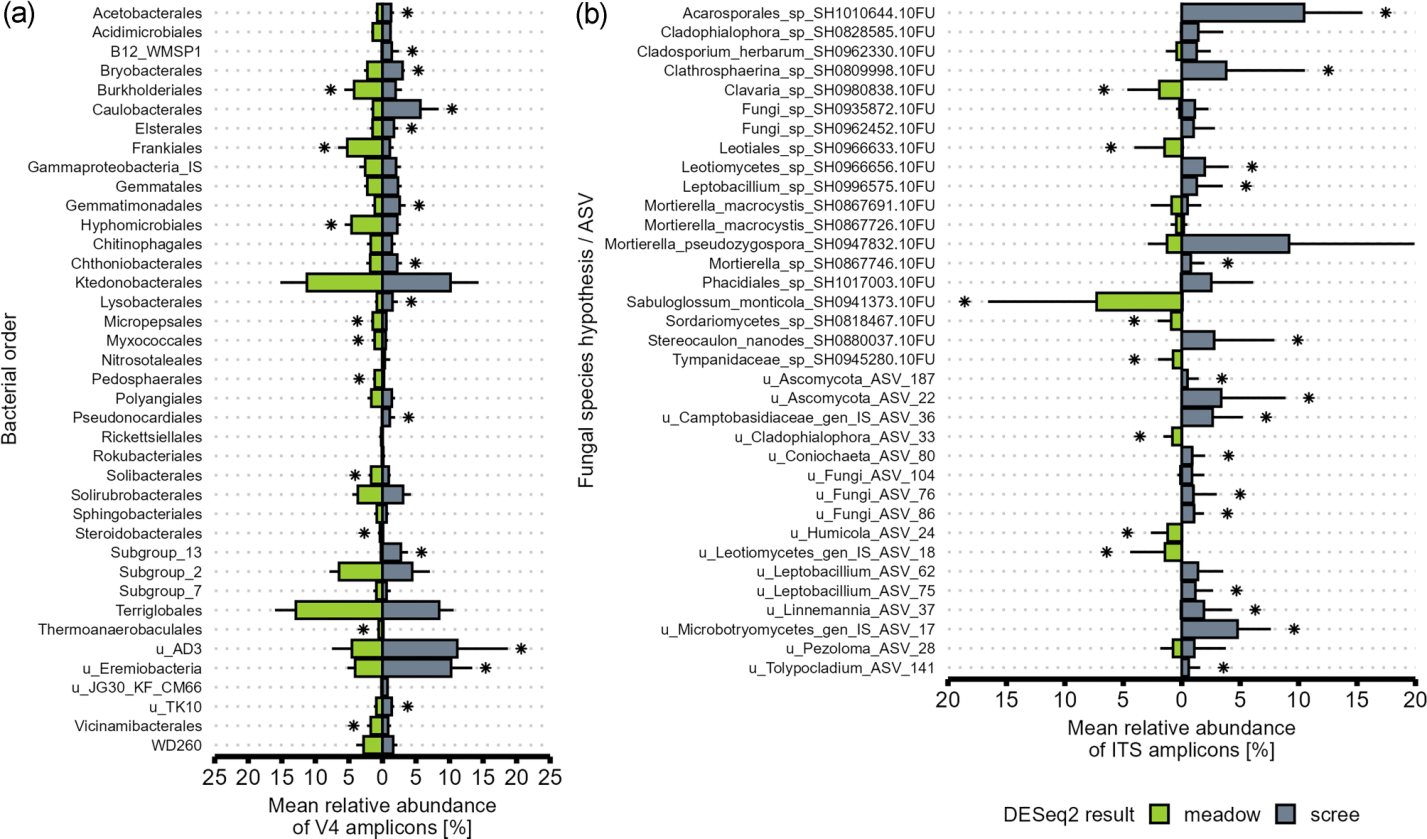
Mean relative abundance of the most abundant bacterial orders (A) and fungal SH/ASVs (B) (≥0.75% in ≥3 samples). Columns represent mean relative abundance, and error bars indicate standard deviation. Column color highlights significantly higher abundance in meadow or scree soils, as determined by DESeq2.

Although the proportion of bacteria in the soil DNA was similar in both soil types, the bacterial communities of the meadows were richer and more diverse (Table S9), and their composition differed markedly from those of the scree soils in all catchments (Fig. 3A, Table S5). Both meadow and scree soils were dominated by Ktedonobacteriales, Terriglobales, Acidobacteria subgroup 2, AD3, and Eremiobacteria (Fig. 4A, Table S6). However, the meadow microbiome was enriched in Burkholderiales, Frankiales (namely *Acidothermus*), Hyphomicrobiales, Solibacterales, Micropepsales, Pedosphaerales, Myxococcales, Vicinamibacterales, Steroidobacterales, and Thermoanaerobaculales. In contrast, scree soils were relatively richer in 13 out of the 39 most abundant bacterial orders, with AD3, Eremiobacteria, Caulobacterales, Acidobacteria subgroup 13, and Pseudonocardiales being the most enriched compared to meadows.

The fungal community composition also differed between the two soil types (Fig. 3B, Table S5). In general, fungal SH/ASV richness in scree soils was about half that in meadow soils (Chao1 ∼89 vs. ∼169; Table S9). Consequently, the sum of the most abundant fungal SH/ASVs (≥0.75% in ≥3 samples) reached ∼60% of all fungal ITS amplicons in scree, but only ∼20% in meadow soils (Fig. 4B).

Among all communities, the most common groups were soil molds of the phylum Mortierellomycota, saprotrophic *Sabuloglossum* sp., lichenized Acrosporales, and several fungal SHs with unclear taxonomic assignment (Fungi *incertae sedis* or Ascomycota *incertae sedis*), although their species composition (SH) differed significantly between scree and meadow soils (Fig. 4B, Table S8). Scree fungal communities were particularly enriched in lichenized lineages (Acarosporales, *Lecanora, Rhizocarpon, Stereocaulon*), which represented a considerable proportion of the scree communities but were negligible in meadow soils. Similar enrichment patterns were observed for saprotrophic *Clathrosphaerina, Leptobacillium, Linnemannia*, and unidentified Microbotryomycetes (mostly yeasts).

In contrast, meadow soils exhibited higher fungal richness and included various saprotrophic guilds that were minor in scree soils: litter saprotrophs (*Gyoerffyella, Hyaloscypha*), soil saprotrophs (*Clavaria, Sabuloglossum*), wood decomposers (*Humicola*), dung saprotrophs, multiple unspecified saprotrophs, as well as plant-associated fungi: root-associated *Archaeorhizomyces*, mycorrhizal Glomeromycota, and various plant pathogens (Table S8).

### Multivariate linkage between microbial traits and nutrient mobility

**Figure 5:**
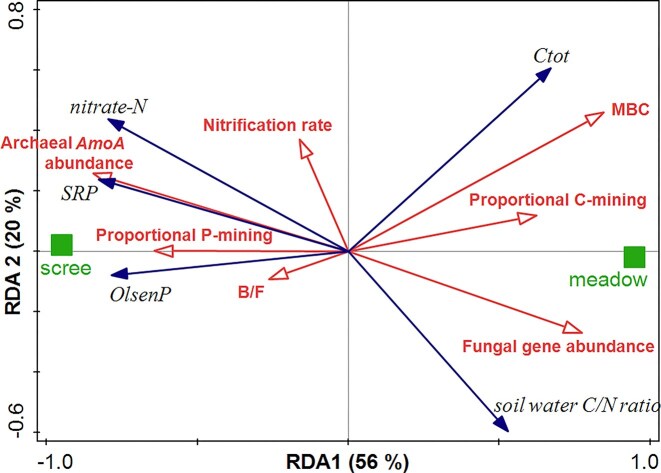
Redundancy analysis (RDA) visualizing multivariate associations between indicators of N and P mobility (response variables) and influential microbial functional traits (explanatory variables) across scree and meadow soils. It shows a clear separation between scree and meadow soils, driven primarily by soil organic C, microbial biomass (MBC), and fungal dominance typical of meadows, as well as indicators of high mineral N and P mobility associated with high abundance of archaeal nitrifiers (archaeal *amoA* gene abundance), high nitrification rate, and high relative investment in P-mining in scree soils.

Redundancy analysis (RDA) used to assess co-variation between microbial traits (in red) and nutrient mobility clearly separated soil types (Fig. 5). Microbial traits associated with nutrient mobilization, such as high archaeal *amoA* gene abundance, high nitrification rate, and greater relative investment in phosphatase activity (which was closely correlated with oxidative activity, though not shown here for figure clarity) consistently aligned with the scree cluster characterized by elevated NO₃⁻-N, SRP, Olsen P, and low C/N of water extract. In contrast, high MBC (overlapping with non-visualized high hydrolytic enzymatic activity), high fungal abundance (18S rRNA gene abundance), and greater investment in C-mining tended toward organic meadow soils with higher nutrient retention potential. The microbial traits used as explanatory variables accounted for 76% of the adjusted explained variation (total model variation of 144.0; permutation test results for all axes yielded a pseudo-F value of 10.3 and a *P* value of 0.001). MBC was the most influential factor, followed by nitrification rate, archaeal *amoA* gene abundance, and fungal abundance (explaining 29.8%, 15.1%, 11.5%, and 5.2% of the adjusted explained variation), with the latter’s influence being insignificant.

## Discussion

### Well established contrasting nutrient pools and mobility in scree vs. meadow soils

Previous studies have shown that two dominant soil habitats in high-elevation catchments—shallow and discontinuous scree soils and vegetation-covered thicker organic rich alpine meadow soils—differ markedly in their ability to release N and P into water bodies (Kaňa et al. 2023, Kopáček et al. 2015a, 2021). We supported these findings by showing disproportionately high concentrations of water-soluble NO₃-N and available P (SRP and Olsen P) in scree soils, along with a limited ability to retain phosphate and higher P leaching measured by field-exposed IER-traps. This pattern identifies scree soils as hotspots for nutrient leaching into waters (Kopáček et al. 2015b). The contrast between scree and meadow soils in alpine ecology is thus well established. The novelty of our study lies not in reiterating these differences, but in demonstrating that, despite the strong inherent covariation of soil development, chemistry, and microbial biomass, the variation in nutrient mobility across these habitats is consistently reflected by microbial traits and functional potentials associated with nutrient mobilization and retention.

### Microbiome composition and functional strategies

To explore mechanisms underlying contrasting nutrient patterns, we examined soil microbiomes in scree and meadow habitats. Both were dominated by bacteria and fungi typical of harsh, oligotrophic, and often acidic environments, including alpine, polar, and permafrost soils (Männistö et al. 2013, Frey et al. 2016, Ji et al. 2017, D’Alò et al. 2022, Holmberg and Jørgensen 2023, Mandolini et al. 2025). Key bacterial taxa included Ktedonobacteriales (Chloroflexi), Terriglobales and Subgroup 2 (Acidobacteriota), and Frankiales (mostly *Acidothermus* sp., Actinobacteriota), while dominant fungi were saprotrophs from Mortierellomycota and Geoglossaceae.

Despite these shared features, scree and meadow microbiomes differed markedly. Scree soils had lower bacterial diversity and a distinct community enriched in candidate phyla AD3 (*Candidatus* Dormibacterota) and Eremiobacteria (formerly WPS-2; *Candidatus* Eremiobacterota), which occur in undeveloped alpine and polar soils and low-organic subsurface horizons (Männistö et al. 2013, Ji et al. 2017, Brewer et al. 2019). These taxa are adapted to oligotrophy through slow growth, spore formation, and the ability to utilize atmospheric gases (e.g. H₂, CO, CO₂) as energy and carbon sources (Ji et al. 2017). Fungal assemblages were dominated by lichenized lineages (e.g. Acarosporales, *Lecanora, Rhizocarpon, Stereocaulon*), pioneer organisms capable of colonizing glacial forefields and bare mineral substrates, tolerant to higher UV radiation, drought, and cold, initiating biological weathering and early carbon accumulation (Coleine et al. 2022, Trejos-Espeleta et al. 2024). Their contribution to nutrient cycling in buried scree soils is likely limited due to limited activity without photobionts (Nash 2008). Nutrient turnover in scree soils is therefore probably driven mainly by saprotrophs recycling microbial necromass, such as *Mortierella, Leptobacillium* or *Linnemannia* (Brabcová et al. 2016, Zare and Gams 2016, De Tender et al. 2024).

In contrast, meadow soils supported larger microbial biomass, promoting stronger immobilization of C, N, and P. Bacterial communities were enriched in Frankiales (notably *Acidothermus*), Solibacterales, Hyphomicrobiales, and Burkholderiales—groups involved in nutrient cycling, organic matter turnover, and plant-root associations. *Acidothermus* species degrade plant polymers under acidic conditions (Ivanova et al. 2016), while Burkholderiales and Hyphomicrobiales include N-fixing and plant-growth-promoting taxa. Acidobacteriota (e.g. Terriglobales, Subgroup 2, Bryobacterales) contribute to the decomposition of complex C sources in alpine soils (Männistö et al. 2013, Zou et al. 2022, Praeg et al. 2025). Fungi were more abundant and functionally diverse, including saprotrophs decomposing plant litter (e.g. *Cadophora, Hyaloscypha, Humicola*), animal excrements (e.g. *Pleuroascus*) (Ludley and Robinson 2008, Plishka et al. 2008, Steindorff et al. 2021), and root-associated taxa (e.g. *Archaeorhizomyces, Gyoerfyella, Humicola, Phialocephalla, Sabuloglossum*). This diversity supports lignin and cellulose decomposition, nutrient cycling, and plant–microbe interactions (Margesin et al. 2009, Rosling et al. 2011, Wang et al. 2017, Chen et al. 2022).

Recent studies show that fungi, more than bacteria, underpin the ecological stability of alpine meadow soils by enhancing long-term nutrient retention and organic matter stabilization (Zou et al. 2022, Li et al. 2024). The high fungal abundance and structural complexity promote slower decomposition and greater accumulation of organic C in microbial biomass and soil organic matter compared to scree soils (Trejos-Espeleta et al. 2024, Whalen et al. 2024, Zhang et al. 2024). This functionally diverse, plant-supported microbial network is central to the high nutrient retention capacity of meadow soils (Van Der Heijden et al. 2006). Arbuscular mycorrhizal Glomeromycota were also detected, but their diversity is underestimated by our primers (Stockinger et al. 2010) and is not discussed further. Plant colonization thus fundamentally restructures the microbiome in alpine systems from oligotrophic, stress-tolerant pioneers to complex, plant-associated assemblages that enhance nutrient retention and carbon stabilization.

### Functional specialization: N and P mobilization potential

Biomass and structural differences in the microbiomes of scree and meadow soils translate into distinct functional potentials for N and P cycling. The small, pioneer-dominated scree microbiome, which exhibits characteristics that promote weathering and nutrient solubilization (Ji et al. 2017, Coleine et al. 2022, Trejos-Espeleta et al. 2024), shows a clear predisposition for efficient key functions in the mobilization of N and P. Scree soils displayed a low genetic capacity for biological N fixation, consistent with long-term increased N input through atmospheric deposition (Kopáček et al. 2015b) but also a low denitrification potential, which would otherwise reduce nitrate concentrations, likely due to limited organic substrate availability and the high oxidative capacity of scree soils. Nevertheless, organic N mining, N mineralization, and nitrification occurred at rates comparable to those in meadow soils with much larger microbiome, i.e. these processes occurred at high biomass-specific rates in scree soils despite their smaller overall microbial biomass. The high abundance of archaeal *AmoA* genes and enrichment of Nitrososphaerales (class Nitrososphaeria, formerly part of Thaumarchaeota) and Nitrosotaleales point to ammonia-oxidizing archaea as key drivers of nitrification, consistent with patterns reported for alpine and permafrost soils (Bates et al. 2011, Siljanen et al. 2019, Zhao et al. 2024). This functional alignment between N availability, nitrification potential, and microbial community structure is further supported by redundancy analysis, which revealed coordinated shifts of scree samples toward high nitrate concentrations, archaeal nitrifiers, and elevated oxidative enzyme and nitrification activities. Overall, these results indicate that scree microbiomes are functionally specialized for N mobilization rather than retention, reflecting an adaptation to their low-organic, high-oxidative environment.

We also found some microbial properties that align with high P mobility and leaching from scree soils. The microbiome in P-rich scree soils showed higher relative investment into phosphatase activity compared to meadows. This likely reflects stress-tolerant adaptations of pioneer microbes, which maintain high enzyme production to maximize phosphate scavenging from transient organic sources (Sinsabaugh et al. 2008, Margesin et al. 2009). Although SRP was high in scree soils, a substantial fraction of P is likely present in microbially derived organic compounds within biofilms and microbial necromass, maintaining demand for phosphatase activity even under high inorganic P availability. High investment in P-mining was coordinated with high oxidase activity, which has been observed in C-limited soils, where microbial communities rely on oxidative mechanisms to decompose complex organic matter and liberate bound nutrients, including P (Schimel and Weintraub 2003, Sinsabaugh et al. 2009). In this context, phosphatases function as a part of a broader enzymatic system that supports nutrient acquisition under oxidative and oligotrophic conditions. Elevated phosphatase activity in scree soils therefore does not necessarily indicate P limitation but instead reflects microbial adaptation to unstable, C-poor environments with episodic nutrient availability.

Despite high P availability, scree soils contained roughly three times fewer P-solubilizing bacteria (PSB; ∼0.3% of the community) than meadow soils. In both soil types, PSB belonged to groups with known P-solubilizing representatives, including *Burkholderia* sensu lato, *Paenibacillus, Pseudomonas, Collimonas, Rahnella*, and *Serratia* (Uroz et al. 2009, Pastore et al. 2020, Rawat et al. 2021). These widespread and versatile taxa, often associated with the rhizosphere, possess adaptations that allow them to persist in oligotrophic, undeveloped alpine soils (Compant et al. 2008, Uroz et al. 2009, Leveau et al. 2010, Huang et al. 2020). The lower PSB abundance in scree soils suggests a reduced genetic potential for P solubilization compared to meadow soils. However, this potential must be interpreted in the context of contrasting environmental conditions: in unstable scree habitats, ongoing physical weathering and downslope transport continually expose fresh mineral surfaces containing apatite. Even small PSB populations can repeatedly exploit these substrates, meaning that dynamic substrate renewal may compensate for lower genetic potential and enhance the effective microbial contribution to phosphate availability.

In contrast, meadow soils support larger microbial biomass, higher fungal abundance, and close coupling with perennial vegetation, enhancing nutrient immobilization in microbial biomass, plant organs persisting through winter and in slowly decomposing organic matter (Körner 2003, Schmidt and Lipson 2004, Bardgett et al. 2007, Kuhnert et al. 2012, Blume-Werry et al. 2016). Nutrients incorporated into litter and humus remain stored for decades due to low temperatures and short growing seasons (Adair et al. 2008, Brooks et al. 2011), while declining C/N ratios in organic horizons indicate progressive nutrient accumulation, primarily driven by long-term N deposition and likely supported by lengthening of the growing season under climate warming (Kopáček et al. 2025). Together, these features reflect the stronger nutrient-retentive function of meadows compared to scree soils, mediated by their plant–microbe–organic matter system.

### Ecological implications for nutrient export to alpine waters

The contrasting microbial structure and function of scree and meadow soils have important consequences for biogeochemical fluxes in alpine catchments. Scree soils are prone to releasing mobile N and P forms, contributing to subtle eutrophication of receiving waters, as evidenced by increasing concentrations of chlorophyll and organic N in alpine lakes in the Tatra Mountains (Kopáček et al. 2024, 2019, 2015a). Our findings confirm that these terrestrial element losses are not solely driven by abiotic factors but also reflect active microbial processes, particularly the strong mobilization capacity of small, specialized microbial communities in scree soils.

In contrast, meadow soils act as more effective biotic buffers, retaining and recycling nutrients through larger microbial and fungal biomass and functional integration with plants. Combined with high soil organic matter content and slow litter decomposition, these traits promote long-term immobilization of N and P in organic pools, thereby reducing nutrient transfer to aquatic ecosystems.

From a broader perspective, our findings suggest that climate change and shifts in vegetation cover may strongly influence nutrient cycling and water quality in alpine landscapes. Intensified physical weathering associated with more frequent freeze–thaw cycles and extreme precipitation events has already been observed in the Tatra Mountains (Kopáček et al. 2021, 2017) and is expected to increase under future climate scenarios (Guo et al. 2024). Prolonged soil exposure due to declining snowpack (Kosolapova and Altshuler 2024), and reduced vegetation growth and nutrient retention during summer droughts (Broadbent et al. 2024) may further enhance nutrient mobilization and leaching from undeveloped soils. In parallel, atmospheric N inputs, which still exceed critical loads in the Tatra Mountains (∼7 ha^−1^ yr^−1^; Kopáček et al. 2015b) and other alpine regions (Rogora et al. 2006, Bowman et al. 2012, Simpson et al. 2014, Kosonen et al. 2019, Balestrini et al. 2024), along with increasing P deposition (Brahney et al. 2015, Scholz and Brahney 2022), could further exacerbate imbalances in nutrient cycling and amplify nutrient export from young alpine soils with low sorption capacity.

Conversely, over the long term, climate change may facilitate vegetation succession and soil development in previously barren alpine zones (Gottfried et al. 2012, Pauli et al. 2012). Expansion of alpine meadows to higher elevations could increase soil microbial biomass and stabilize nutrient fluxes through organic matter accumulation and plant–microbe interactions (Bardgett and Walker 2004, Nemergut et al. 2005), partially offsetting the negative effects of increasing erosion and weathering in scree areas and the associated nutrient leaching.

## Conclusion

Our study reveals that the fundamental differences in the ability of scree and meadow soils to retain or release nitrogen and phosphorus are driven not only by soil developmental stage and nutrient stocks but also by distinct microbial communities. We extend existing alpine soil studies by demonstrating a coordinated alignment between microbial functional traits and nutrient mobility in a natural system where soil age, chemistry and biology cannot be experimentally disentangled. In organic-poor scree soils, small pioneer microbiomes exhibit high biomass-specific activities that promote nutrient mobilization and potential leaching. In contrast, meadow soils, enriched in fungal biomass and influenced by plant–microbe interactions, act as robust nutrient reservoirs, promoting retention and recycling. These results provide strong evidence for a microbial footprint in regulating nutrient fluxes and highlight the central role of soil microbiota in determining the character and openness of nutrient cycles. As climate change alters alpine ecosystems, preserving alpine meadows will be key to sustaining long-term nutrient retention and water quality.

## Supplementary Material

fiag008_Supplemental_File

## Data Availability

Sequencing data, comprising soil microbiome amplicon reads, and 16S rRNA gene sequences of phosphorus solubilizing bacterial isolates, are publicly available in the European Nucleotide Archive (ENA) under study accession PRJEB96838. Isolate sequences are accessible under accession numbers OZ335379–OZ335415.
